# Dental Exposure and Oral-Flora-Associated Infective Endocarditis: A Transoesophageal Echocardiography-Based Study at a Teaching District General Hospital in the United Kingdom

**DOI:** 10.7759/cureus.114994

**Published:** 2026-08-22

**Authors:** Abdelhalim Eltaib, Bushra Ahmed, Mueedudin Akram, Maryam Kazanji, Ho Yau Chloe Vun, Areej Hussien, Mohammad Mohammad, Surojit Bose

**Affiliations:** 1 Cardiology, University Hospitals of Derby and Burton NHS Foundation Trust, Derby, GBR; 2 Internal Medicine, University Hospitals of Derby and Burton NHS Foundation Trust, Derby, GBR; 3 Psychiatry, University Hospitals of Derby and Burton NHS Foundation Trust, Derby, GBR

**Keywords:** aortic valve infective endocarditis, culture-negative endocarditis, infective endocarditis, lead-associated vegetation, prosthetic valve infective endocarditis, transoesophageal echocardiography

## Abstract

Background

Infective endocarditis (IE) remains associated with substantial morbidity and mortality despite advances in diagnosis and treatment. Transoesophageal echocardiography (TOE) is central to the diagnosis of IE; however, contemporary data examining the relationships among dental exposure, microbiological findings, and echocardiographic characteristics in routine clinical practice remain limited, particularly in district general hospitals (DGHs).

Methods

We conducted a retrospective observational study of consecutive TOE examinations performed for suspected IE at Royal Derby Hospital, a United Kingdom (UK) teaching DGH, between April 2022 and October 2024. A total of 145 consecutive TOE examinations performed for suspected IE were screened. Twelve examinations were excluded because IE was not confirmed or because they were duplicate or repeat examinations relating to the same infective episode, leaving 133 unique confirmed clinical episodes of IE for the final analysis. Demographic, microbiological, echocardiographic, treatment, and outcome data were collected retrospectively. The primary analysis evaluated the association between documented dental procedures and oral flora-associated IE, while secondary analyses described echocardiographic findings, microbiological profiles, surgical intervention, and in-hospital mortality.

Results

Among the 133 unique confirmed clinical episodes of IEepisodes, the mean patient age was 63.3 years, and 66.2% of patients were male. Native-valve IE accounted for 75.9% of cases. The aortic valve was the most frequently affected structure (62.4%), while *Staphylococcus aureus* was the predominant causative organism (30.8%). Oral flora organisms accounted for 33.1% of infections. Twenty-six patients (19.5%) underwent surgical or device intervention, and the in-hospital mortality rate was 11.3%. Among the 26 patients with documented dental procedures, antibiotic prophylaxis was documented for two (7.7%). Documented dental procedures were associated with a higher frequency of oral flora-associated IE (50.0% vs. 28.8%; odds ratio, 2.47; 95% confidence interval, 1.03-5.94; p = 0.040). No clinical, microbiological, or echocardiographic variable demonstrated a significant association with in-hospital mortality.

Conclusions

In this contemporary cohort of patients with confirmed IE who underwent TOE, documented dental exposure was significantly associated with oral flora-associated IE. The study also demonstrates that aortic valve involvement, *Staphylococcus aureus* infection, and native-valve disease remain common in routine UK practice. The low rate of documented antibiotic prophylaxis may highlight an opportunity to improve documentation, although prospective studies are needed to determine the true rate of prophylaxis administration.

## Introduction

Infective endocarditis (IE) remains a life-threatening disease associated with substantial morbidity and mortality despite advances in antimicrobial therapy, multimodality imaging, and cardiac surgery. The epidemiology of IE has changed substantially over recent decades. In England, the incidence remained relatively stable at approximately 27 cases per million population per year until 2009 but subsequently increased to around 50 cases per million population per year by 2018, representing an 86% rise. Despite advances in diagnosis and treatment, one-year mortality continues to exceed 30%, highlighting the persistent burden of this disease [1-6].

Transoesophageal echocardiography (TOE) is a cornerstone of the diagnostic evaluation of suspected IE and constitutes a major imaging component of the modified Duke criteria. Compared with transthoracic echocardiography, TOE provides superior sensitivity for detecting valvular vegetations, peri-annular abscesses, prosthetic valve infection, leaflet perforation, and cardiac implantable electronic device (CIED)-related infection. Consequently, current European and American guidelines recommend TOE in patients with suspected IE when clinical suspicion remains high, particularly in the presence of prosthetic valves, intracardiac devices, or inconclusive transthoracic echocardiographic findings [1,3,7-9].

The microbiological profile of IE has also evolved, with *Staphylococcus aureus* emerging as the predominant pathogen in many contemporary cohorts, while viridans group streptococci and enterococci continue to account for a substantial proportion of cases. Oral microorganisms remain important causative pathogens, particularly in patients with underlying structural heart disease or prosthetic valves. Good oral hygiene and appropriate dental care are therefore recognised as important preventive measures. Current international guidelines recommend antibiotic prophylaxis only for patients at the highest risk of adverse outcomes who are undergoing selected invasive dental procedures [1-6,10-15].

Although several studies have described the microbiological and echocardiographic characteristics of IE, relatively few have specifically evaluated the relationship between documented dental exposure and oral flora-associated IE in routine clinical practice. Furthermore, data from UK teaching district general hospitals (DGHs) remain limited, particularly regarding the documentation of antibiotic prophylaxis administration and recent dental procedures. A better understanding of these associations may help improve risk assessment, reinforce collaboration between cardiology and dental services, and identify opportunities to strengthen preventive strategies for patients at increased risk of IE [14,15].

The primary aim of this retrospective observational study was to evaluate the association between documented dental procedures and oral flora-associated IE among patients with confirmed IE who underwent TOE at a UK teaching DGH. The secondary objectives were to describe the clinical, microbiological, and echocardiographic characteristics of the cohort, evaluate surgical intervention and in-hospital mortality, and assess the documentation of antibiotic prophylaxis in patients with recent dental procedures. This study was conducted within the UK clinical context, where National Institute for Health and Care Excellence (NICE) guidance (CG64) states that antibiotic prophylaxis is not routinely recommended for patients undergoing dental procedures.

## Materials and methods

Study design and setting

This retrospective observational study was conducted at Royal Derby Hospital, a UK teaching DGH providing secondary and tertiary cardiology services. Consecutive adult patients who underwent TOE for suspected IE between April 2022 and October 2024 were identified from the departmental echocardiography database. Clinical records, microbiology results, imaging studies, operative reports, and electronic medical records were reviewed retrospectively. The diagnosis of IE was established using the modified Duke criteria in conjunction with multidisciplinary clinical assessment, microbiological findings, and echocardiographic evidence, in accordance with contemporary international guidelines [1,7].

Study population

A total of 145 consecutive TOE examinations performed for suspected IE were screened. Examinations relating to cases in which IE was not confirmed and duplicate or repeat examinations relating to the same infective episode were excluded. The final study cohort consisted of 133 unique confirmed clinical episodes of IE.

Patients aged 18 years or older with confirmed IE who underwent TOE during the study period were eligible for inclusion. No cases classified as possible IE were included in the final cohort. Repeat TOE examinations performed during the same infective episode were considered part of a single clinical episode and were not analysed separately.

As this was a retrospective observational study that included all consecutive eligible patients during the study period, no formal a priori sample size calculation was performed.

Data collection

Patient demographics, cardiovascular risk factors, predisposing cardiac conditions, microbiological results, echocardiographic findings, treatment strategies, and clinical outcomes were extracted from electronic health records. Data extraction was performed using the hospital electronic medical record system and departmental echocardiography database. Where discrepancies were identified, the records were reviewed manually to ensure data accuracy.

The variables collected were age; sex; pre-existing valvular heart disease; prosthetic heart valves; CIEDs; a history of previous IE; blood culture results; causative microorganisms; the affected cardiac valve or intracardiac structure; the presence of vegetations; peri-annular abscess formation; prosthetic valve involvement; device-related infection; the requirement for cardiac surgery or device extraction; and in-hospital mortality.

Echocardiographic assessment

All TOE examinations were performed according to departmental protocols by experienced consultant cardiologists with expertise in TOE and IE.

The recorded TOE findings included vegetation location and size, valve involvement, leaflet perforation, abscess formation, prosthetic valve infection, paravalvular complications, and evidence of CIED infection.

Dental exposure and antibiotic prophylaxis

Dental history was obtained from the electronic medical records and, where patients were alive and contactable, through direct telephone contact. Patients without documented dental exposure in the available records or patient history were classified as having no documented dental exposure. Dental exposure was defined as documentation of an invasive dental procedure involving manipulation of the gingival tissues or periapical region of the teeth or perforation of the oral mucosa within the six months preceding the diagnosis of IE.

Documentation of antibiotic prophylaxis before dental procedures was also recorded where available. Causative microorganisms were classified as oral flora organisms if blood cultures yielded viridans group streptococci, anginosus group streptococci, *Streptococcus pneumoniae*, *Enterococcus faecalis*, or *Streptococcus gallolyticus*/*S. bovis*. *E. faecalis* and *S. gallolyticus*/*S. bovis* were included because of their documented oral carriage, although both also colonise the gastrointestinal tract.

Study outcomes

The primary outcome was the association between documented recent dental procedures and oral flora-associated IE.

The secondary outcomes included the microbiological profile, echocardiographic characteristics, distribution of native- and prosthetic-valve infections, requirement for cardiac surgery or device extraction, in-hospital mortality, and documentation of antibiotic prophylaxis before dental procedures.

Statistical analysis

Continuous variables are presented as the mean ± SD or median with IQR, depending on the data distribution. Categorical variables are expressed as frequencies and percentages.

Comparisons between categorical variables were performed using the chi-square test or Fisher’s exact test, as appropriate. Continuous variables were compared using Student’s t-test or the Mann-Whitney U test, according to the data distribution.

ORs with 95% CIs were calculated to evaluate the association between documented dental exposure and oral flora-associated IE. Statistical significance was defined as a two-sided p-value of <0.05. Statistical analyses were performed using IBM SPSS Statistics, version 30 (IBM Corp., Armonk, NY, USA). Given the relatively small number of oral flora-associated IE cases (n = 44), multivariable logistic regression was not performed because the inclusion of multiple covariates could have resulted in overfitting and unstable estimates. The primary association was therefore assessed using an unadjusted odds ratio with a 95% CI.

Ethical considerations

This study was conducted as a retrospective observational service evaluation using routinely collected clinical data. All patient information was anonymised before analysis in accordance with institutional information governance policies. Ethical approval and individual informed consent were waived in accordance with local governance requirements for retrospective service evaluations.

## Results

Study population

Between April 2022 and October 2024, 145 consecutive TOE examinations performed for suspected IE were screened. Twelve examinations were excluded because IE was not confirmed or because they were duplicate or repeat examinations relating to the same infective episode, leaving 133 unique confirmed clinical episodes of IE for the final analysis. Overall in-hospital mortality during the index admission was 11.3% (15/133).

Baseline characteristics

**Figure 1:**
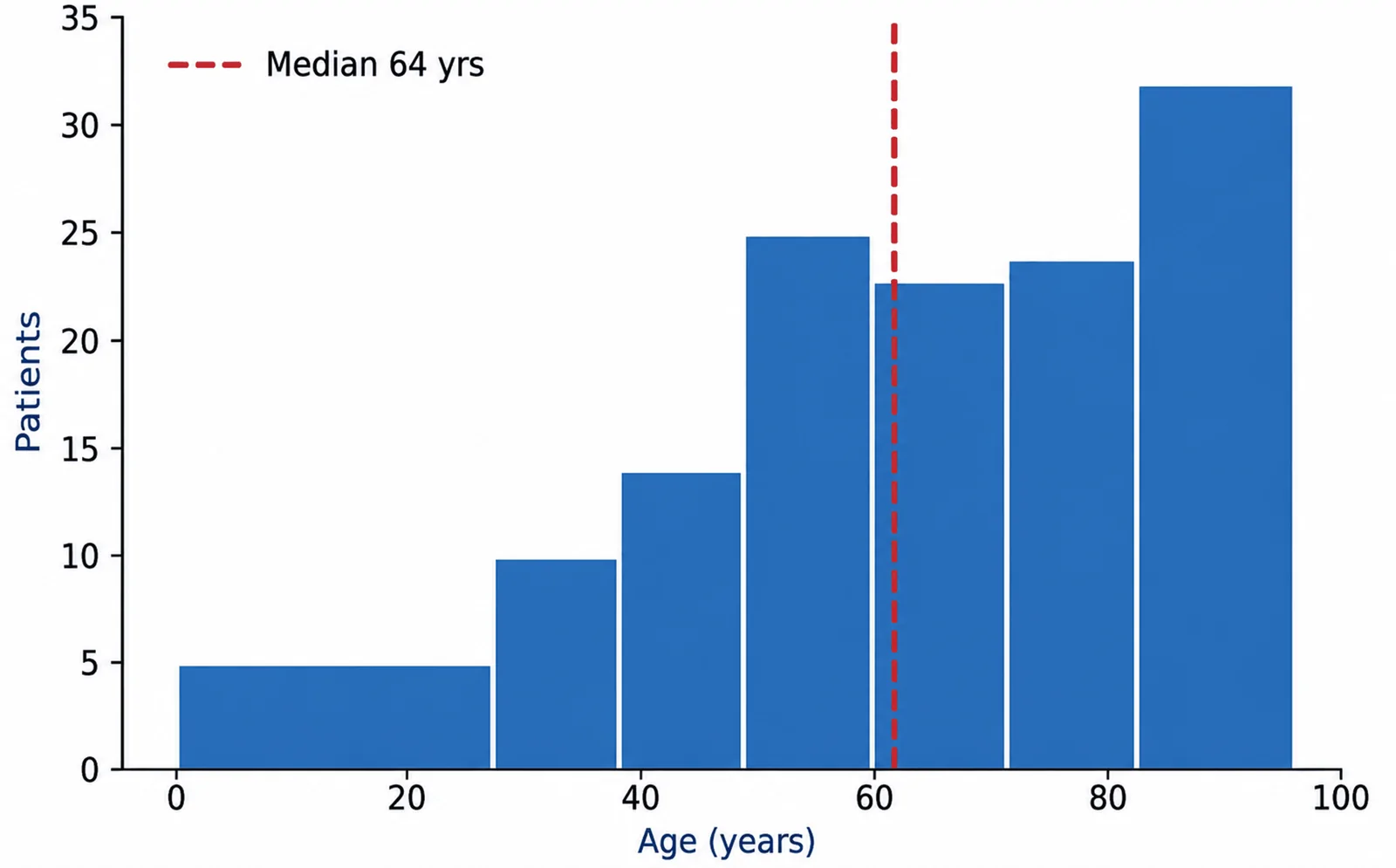
Demographics. Age, mean ± SD: 63.3 ± 17.7 years.

The mean age of the study population was 63.3 ± 17.7 years (median, 64 years; interquartile range, 52-79 years), and 88 patients (66.2%) were male. The age distribution of the study population is shown in Figure 1. Native valve IE accounted for 75.9% of cases, whereas prosthetic valve involvement was identified in approximately one-quarter of patients. The aortic valve was the most frequently affected cardiac structure (62.4%). Intravenous drug use was documented in 9.8% of cases, and no deaths occurred within this subgroup. CIED-related infection was identified in 21 patients (15.8%).

Microbiological findings

**Figure 2:**
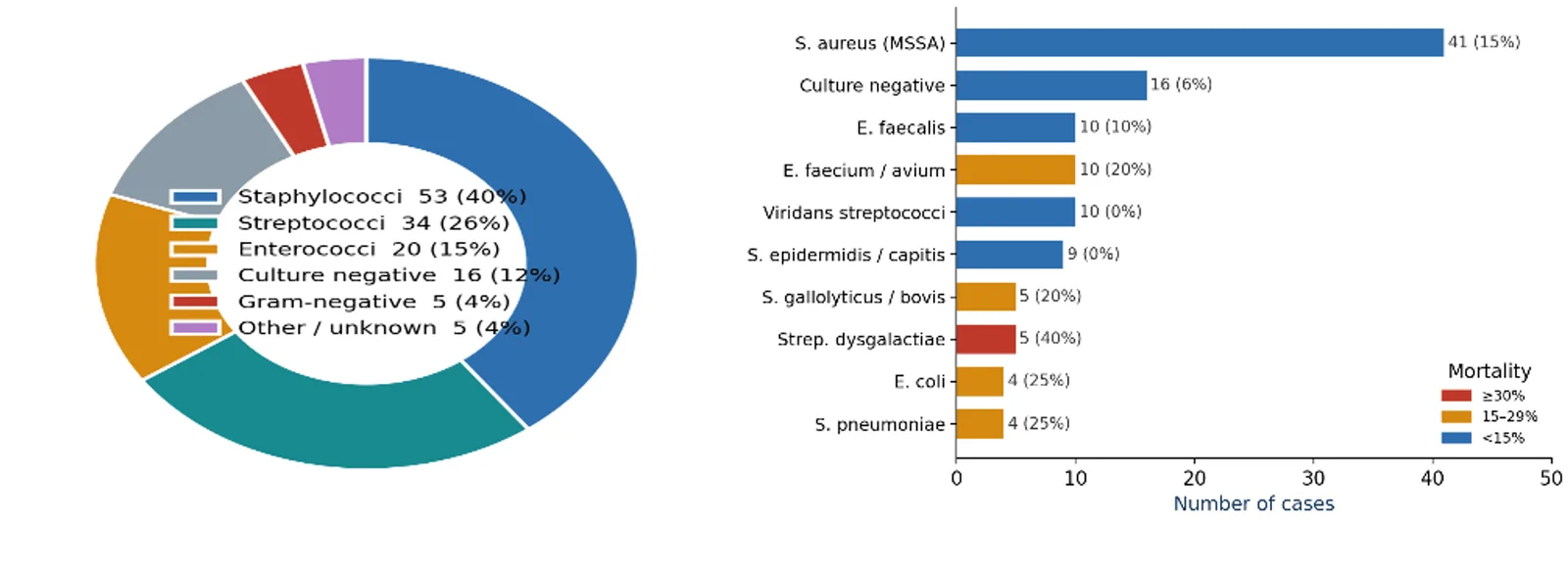
Microbiological distribution. Staphylococcus aureus was the most common organism (30.8%). Streptococci (25.6%) and enterococci (15.0%) reflect a substantial oral/gastrointestinal contribution. Culture-negative infective endocarditis occurred in 12.0% of cases.

*Staphylococcus aureus* was the predominant causative organism, accounting for 30.8% of infections. Streptococcal species represented 25.6% of cases, while enterococci accounted for 15.0%. Oral-flora organisms were identified in 44 patients (33.1%), representing approximately one-third of all episodes. Culture-negative IE occurred in 12.0% of cases. The microbiological distribution is illustrated in Figure 2.

Echocardiographic findings and management

TOE demonstrated that the aortic valve was the most commonly affected valve (62.4%), followed by the mitral valve. Multi-valve involvement was present in 14.3% of patients. Device-related IE accounted for 21 cases (15.8%).

**Figure 3:**
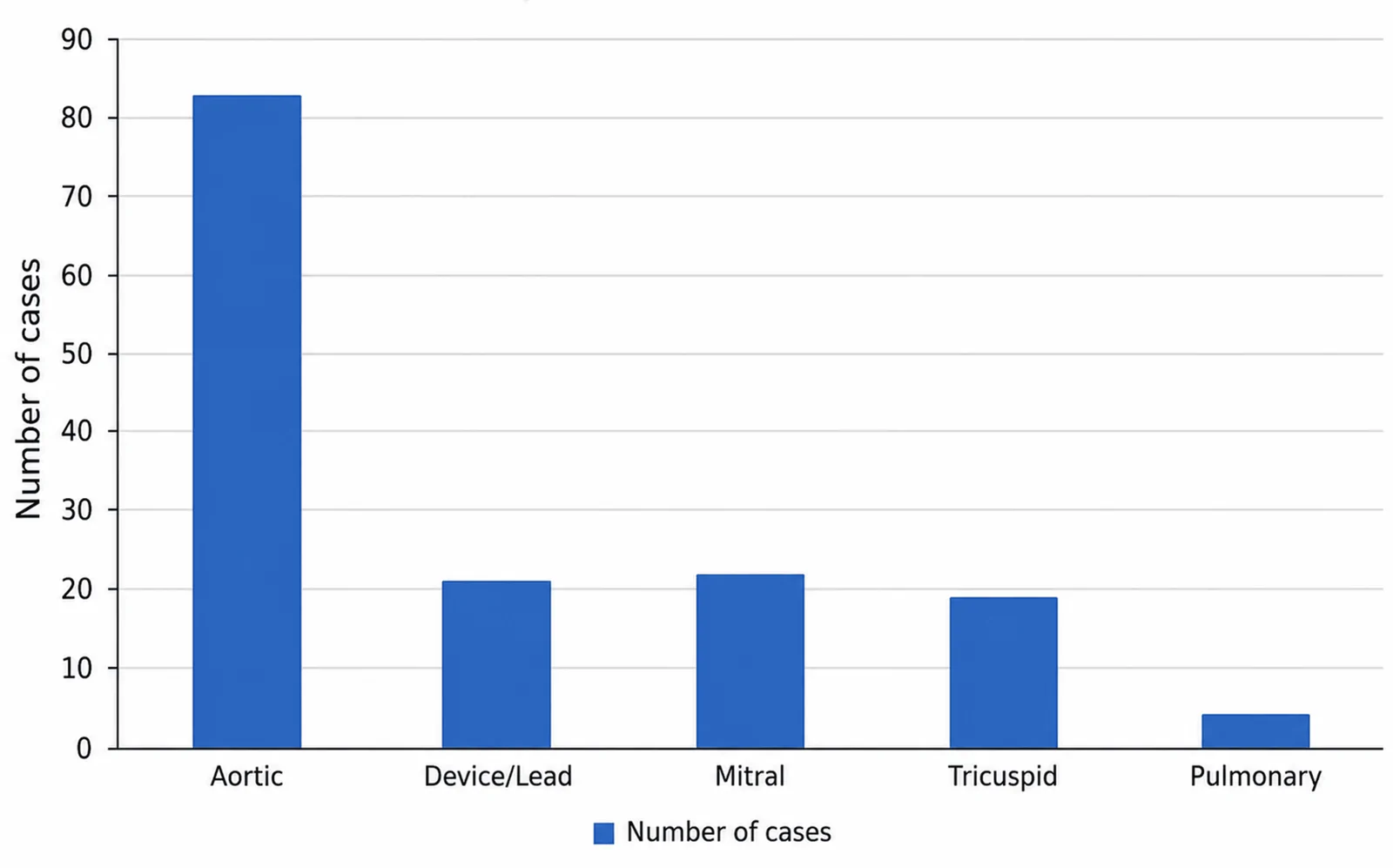
Valve/structure involvement. Multi-valve involvement occurred in 14.3% of patients. Surgical management was undertaken in 26 patients: 17 valve procedures and nine device extractions. In-hospital mortality was 11.3%.

**Figure 4:**
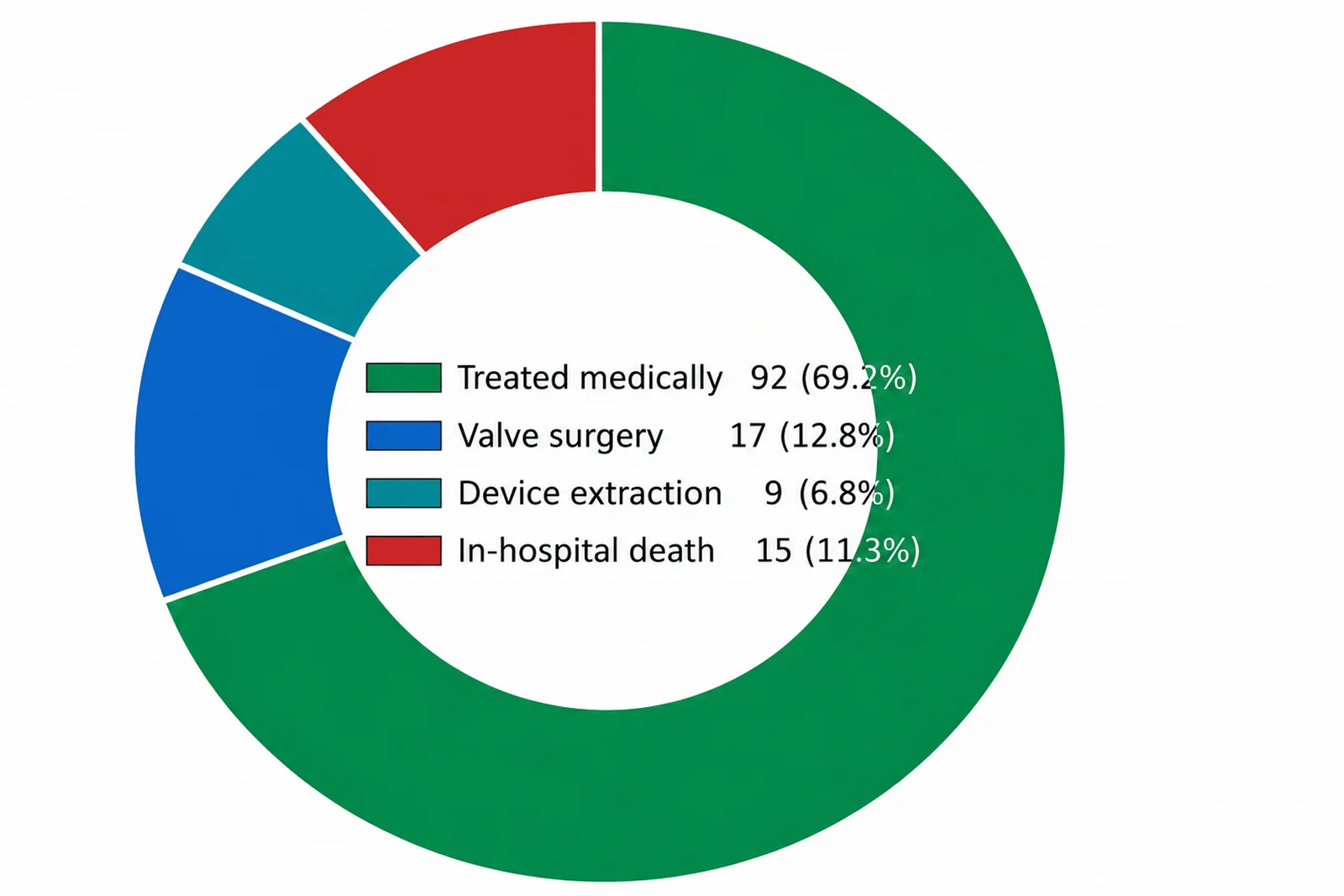
Outcomes. Multi-valve involvement occurred in 14.3% of patients. Surgical management was undertaken in 26 patients: 17 valve procedures and nine device extractions. In-hospital mortality was 11.3%.

Overall, 26 patients (19.5%) underwent an interventional procedure during the index admission, including 17 valve operations and nine device extractions. Among the valve procedures, aortic valve replacement was the most common operation (n = 10), followed by mitral valve replacement (n = 3), tricuspid valve replacement (n = 2), transcatheter aortic valve implantation (TAVI) (n = 1), and valve-in-valve TAVI (n = 1). The distribution of valve and structure involvement is illustrated in Figure 3. Clinical outcomes are summarised in Figure 4.

Association between dental exposure and oral-flora IE

Documented invasive dental procedures before the diagnosis of IE were identified in 26 patients (19.5%). Oral-flora organisms were isolated in 13 of these patients (50.0%), compared with 31 of 107 patients (28.8%) without documented dental exposure.

**Figure 5:**
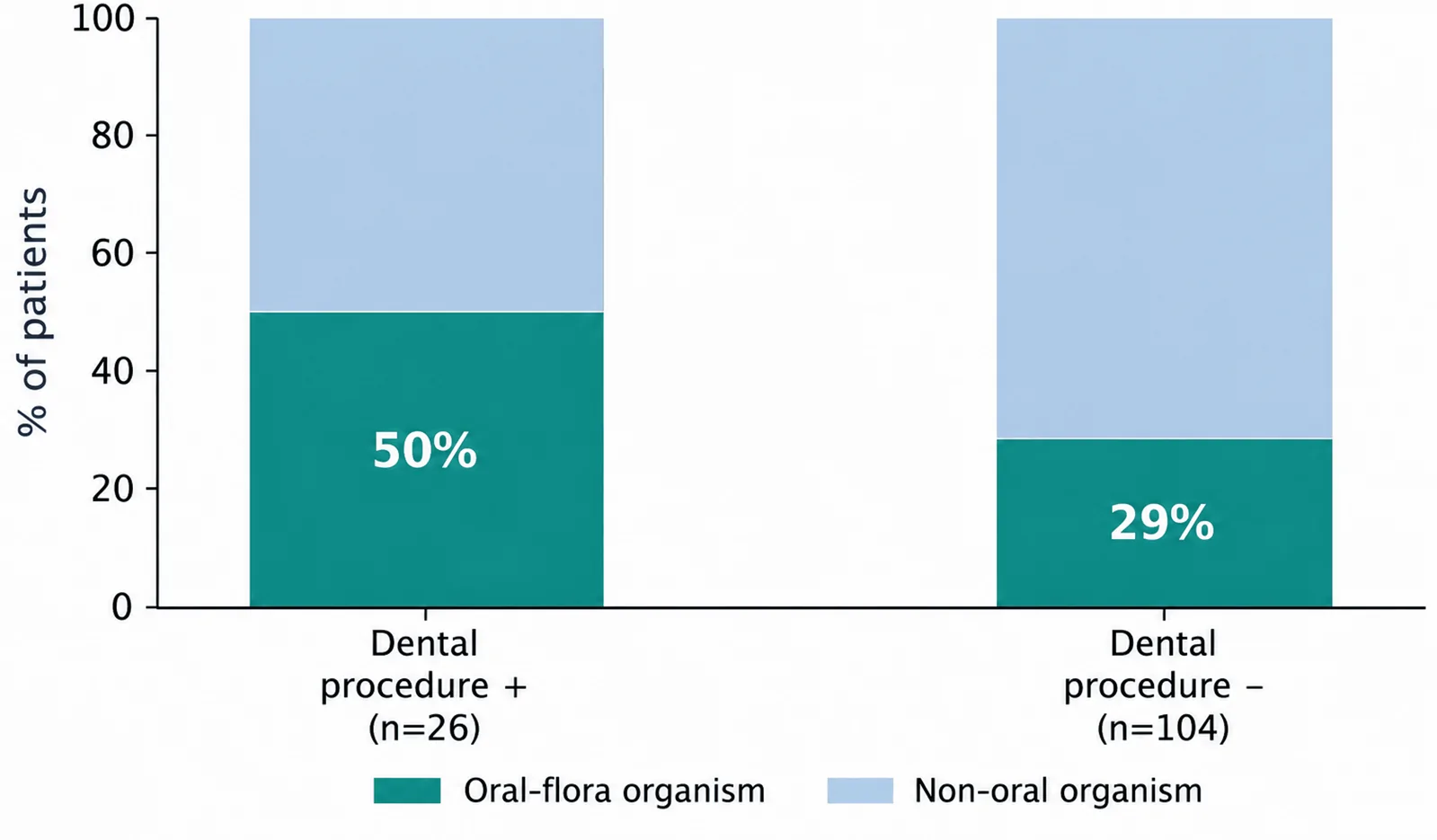
Association between documented dental exposure and oral-flora infective endocarditis. OR = 2.47 (95% CI: 1.03-5.94; p = 0.040). Oral-flora infective endocarditis occurred in 50.0% of patients with documented dental exposure, compared with 28.8% of patients without documented dental exposure.

Patients with documented dental procedures had significantly higher odds of oral-flora IE than those without documented dental exposure (odds ratio, 2.47; 95% CI, 1.03-5.94; p = 0.040). Antibiotic prophylaxis was documented in 2 of 26 patients (7.7%). Among patients with documented dental procedures, prophylaxis status was not documented in 22 of 26 cases (84.6%). These findings are illustrated in Figure 5.

Clinical outcomes

**Figure 6:**
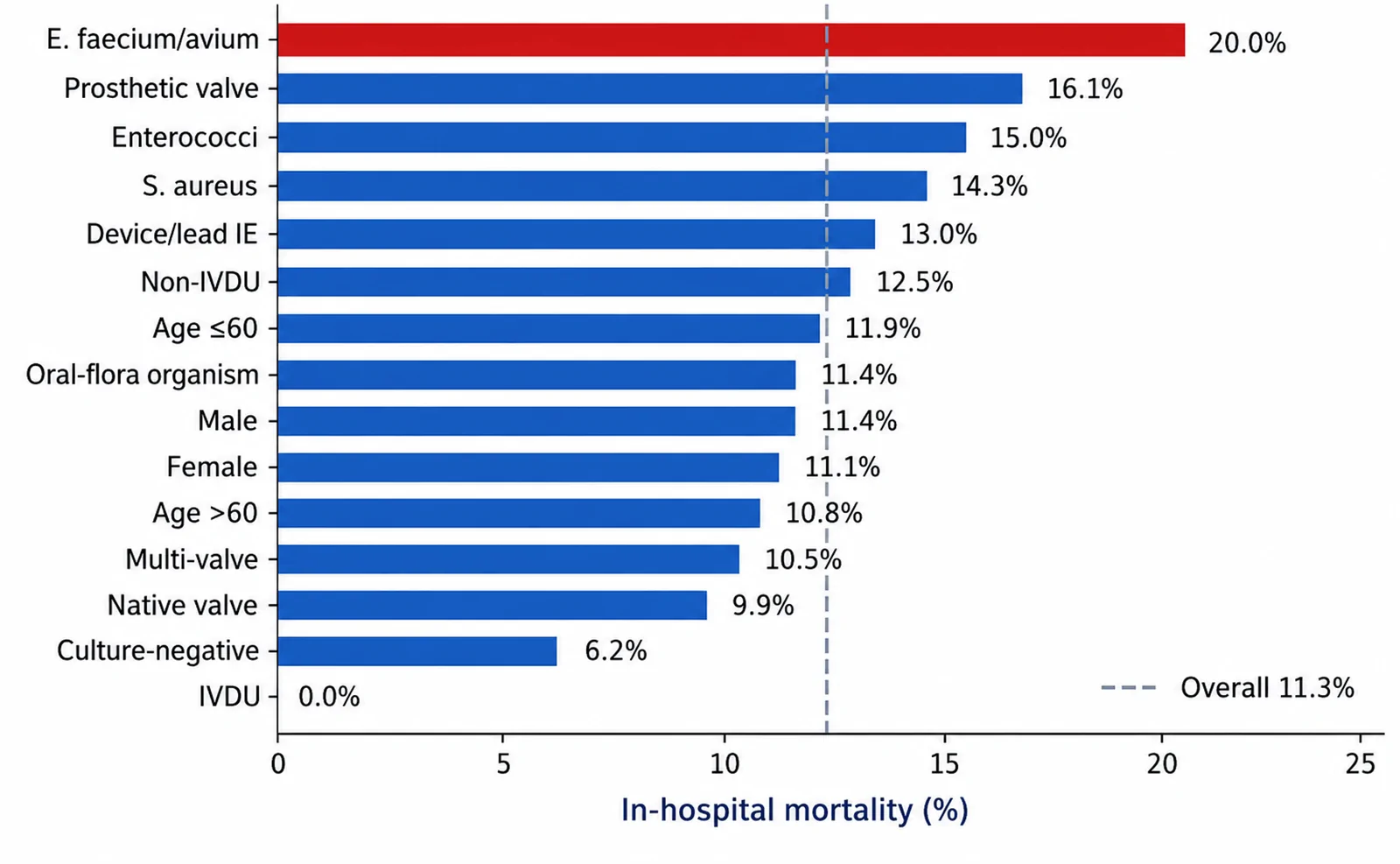
In-hospital mortality. Overall in-hospital mortality was 11.3%, lower than that reported in most published series, reflecting strict in-hospital attribution. Prosthetic valve infective endocarditis had the highest crude mortality (16.1%), but the CIs were wide. No deaths occurred among patients with intravenous drug use (IVDU) (0/13), consistent with their younger age and right-sided disease. Oral-flora infective endocarditis was not associated with excess mortality (11.4%).

Overall in-hospital mortality was 11.3% (15/133). In-hospital mortality was defined as death occurring during the index hospital admission for IE. Prosthetic valve IE had the highest crude mortality (16.1%), although the CIs were wide. No deaths occurred among patients with intravenous drug use, while oral-flora IE was not associated with excess in-hospital mortality (11.4%). No demographic, microbiological, or echocardiographic variable was significantly associated with in-hospital mortality. Mortality across the principal clinical subgroups is illustrated in Figure 6.

## Discussion

This retrospective observational study describes the clinical, microbiological, and echocardiographic characteristics of 133 unique confirmed clinical episodes IE managed at a UK teaching DGH. The principal finding was a significant association between documented invasive dental procedures and oral-flora IE, with patients with documented recent dental exposure demonstrating approximately 2.5-fold greater odds of infection with oral microorganisms. In addition, *Staphylococcus aureus* remained the predominant pathogen, native-valve IE accounted for most cases, the aortic valve was the most frequently affected cardiac structure, and overall in-hospital mortality was 11.3%. These findings provide contemporary data describing the epidemiology of IE in routine clinical practice while highlighting the importance of documenting dental history and oral health in patients presenting with suspected IE [4-6].

The observed association between documented dental exposure and oral-flora IE is biologically plausible and consistent with the recognised pathogenesis of viridans streptococcal endocarditis. Manipulation of gingival tissues during invasive dental procedures may result in transient bacteraemia, particularly in individuals with poor oral hygiene or underlying structural heart disease. However, transient bacteraemia may also occur during routine daily activities such as tooth brushing and chewing, reinforcing the importance of maintaining good oral health in patients at increased risk of IE. Current European Society of Cardiology (ESC) and American Heart Association (AHA) guidelines recommend antibiotic prophylaxis only for carefully selected high-risk patients undergoing invasive dental procedures, whereas National Institute for Health and Care Excellence (NICE) guidance does not recommend routine antibiotic prophylaxis for dental procedures. All three place greater emphasis on preventive dental care and oral hygiene than on widespread antibiotic use [1-3,12-15].

Although our findings demonstrate a statistically significant association between documented dental procedures and oral-flora IE, they should not be interpreted as evidence of causality. Approximately half of the patients with documented dental procedures had IE caused by non-oral organisms, while many patients with oral-flora IE had no recorded recent dental procedure. These findings suggest that incomplete documentation, unrecognised dental disease, spontaneous bacteraemia arising from routine daily activities, or alternative portals of microbial entry may all contribute to the development of IE. Similar observations have been reported in previous studies evaluating the relationship between dental procedures and IE [12-15].

Among the 26 patients with documented dental procedures, prophylaxis was documented in only two patients (7.7%), while 22 patients (84.6%) had no recorded information regarding antibiotic prophylaxis. This may reflect incomplete documentation rather than confirmed non-adherence to guideline recommendations, as this retrospective study cannot determine whether prophylaxis was administered but not recorded or was not administered. Nevertheless, this represents an important quality improvement opportunity. Introducing a standardised IE admission pro forma incorporating mandatory documentation of recent dental procedures, oral health status, and antibiotic prophylaxis may improve adherence to guideline-based practice and facilitate future clinical audits [1-3,14,15].

The microbiological findings observed in this study are consistent with those reported in contemporary international cohorts. *Staphylococcus aureus* remained the most frequently isolated organism, reflecting the increasing contribution of healthcare-associated infection and invasive medical procedures to the epidemiology of IE. Oral-flora organisms accounted for approximately one-third of cases, while enterococci also represented an important proportion of infections, emphasising the heterogeneous microbiological profile of modern IE. These findings are broadly consistent with reports from the International Collaboration on Endocarditis and other contemporary series [4-6,10,11].

Our echocardiographic findings similarly reflect current epidemiological trends. Native-valve IE predominated, with the aortic valve representing the most frequently involved cardiac structure. Device-related IE accounted for a substantial proportion of cases, illustrating the growing burden of infections involving cardiac implantable electronic devices. Approximately one in five patients required surgical intervention or device extraction, emphasising the complexity of managing IE and the importance of multidisciplinary collaboration involving cardiology, cardiothoracic surgery, microbiology, infectious diseases, and cardiac imaging specialists [1,4,5,16-18].

Overall in-hospital mortality was 11.3%, which is lower than that reported in many contemporary registries. This may reflect the use of a strictly defined in-hospital mortality endpoint rather than longer-term follow-up, as well as differences in patient populations and referral pathways. Although prosthetic valve IE had the highest crude mortality, no demographic, microbiological, or echocardiographic variable was significantly associated with in-hospital mortality in this cohort. The relatively small number of mortality events limited the statistical power for multivariable modelling and should be considered when interpreting these findings [4-6,19-21].

This study has several strengths. It includes a contemporary cohort of consecutive confirmed IE episodes identified among patients undergoing TOE for suspected IE at a UK teaching DGH and provides comprehensive clinical, microbiological, echocardiographic, and outcome data. Furthermore, to our knowledge, it is among the few UK studies to specifically evaluate the relationship between documented dental exposure and oral-flora IE while also assessing antibiotic prophylaxis documentation. These findings have direct relevance to clinical practice and quality improvement initiatives.

Several limitations should be acknowledged. First, this was a retrospective single-centre study of patients undergoing TOE for suspected IE, which may introduce selection bias and limit generalisability, as patients who did not undergo TOE, including those who were too unwell for the procedure or were diagnosed through other pathways, were not captured. Second, documentation of dental procedures, their timing and indication, and antibiotic prophylaxis was incomplete, introducing potential information bias. The six-month exposure window was selected to capture recent documented dental exposure, but the broad definition and limited documentation of the timing and nature of individual procedures may have resulted in exposure misclassification. Reverse causation is also possible, whereby patients with undiagnosed IE may develop dental or oral symptoms and seek dental care before the diagnosis of IE is established. Third, the relatively small number of oral-flora-associated IE cases limited the ability to perform reliable multivariable adjustment, and the confidence interval around the primary association was relatively wide (OR, 2.47; 95% CI, 1.03-5.94), indicating uncertainty regarding the precision of the estimate. Fourth, although prophylaxis was documented in only a small proportion of patients, the retrospective design does not allow distinction between prophylaxis that was not administered and prophylaxis that was administered but not documented. Finally, the single-centre DGH setting may limit generalisability to tertiary referral centres and other healthcare settings. Despite these limitations, the study provides contemporary observational data on documented dental exposure and oral-flora-associated IE in a UK teaching DGH [1,4,5,20,21].

## Conclusions

In summary, this study characterises the contemporary presentation of IE within a UK teaching district general hospital. Documented recent dental procedures were associated with oral-flora IE, while *Staphylococcus aureus* remained the leading pathogen. The findings may highlight opportunities to improve the documentation of dental history and antibiotic prophylaxis as part of multidisciplinary preventive care. Prospective multicentre studies are required to determine whether these observations are reproducible in larger populations and to establish the actual rate of antibiotic prophylaxis administration.

## References

[1] Delgado V, Ajmone MN, de Waha S (2023). 2023 ESC Guidelines for the management of endocarditis. Eur Heart J.

[2] Dayer MJ, Jones S, Prendergast B, Baddour LM, Lockhart PB, Thornhill MH (2015). Incidence of infective endocarditis in England, 2000-13: a secular trend, interrupted time-series analysis. Lancet.

[3] (2026). National Institute for Health and Care Excellence. Prophylaxis against infective endocarditis: antimicrobial prophylaxis against infective endocarditis in adults and children undergoing interventional procedures. https://www.nice.org.uk/guidance/cg64.

[4] Wilson W, Taubert KA, Gewitz M (2007). Prevention of infective endocarditis: guidelines from the American Heart Association: a guideline from the American Heart Association Rheumatic Fever, Endocarditis, and Kawasaki Disease Committee, Council on Cardiovascular Disease in the Young, and the Council on Clinical Cardiology, Council on Cardiovascular Surgery and Anesthesia, and the Quality of Care and Outcomes Research Interdisciplinary Working Group. Circulation.

[5] Cahill TJ, Prendergast BD (2016). Infective endocarditis. Lancet.

[6] Holland TL, Baddour LM, Bayer AS, Hoen B, Miro JM, Fowler VG Jr (2016). Infective endocarditis. Nat Rev Dis Primers.

[7] Murdoch DR, Corey GR, Hoen B (2009). Clinical presentation, etiology, and outcome of infective endocarditis in the 21st century: the International Collaboration on Endocarditis-Prospective Cohort Study. Arch Intern Med.

[8] Li JS, Sexton DJ, Mick N (2000). Proposed modifications to the Duke criteria for the diagnosis of infective endocarditis. Clin Infect Dis.

[9] Habib G, Lancellotti P, Antunes MJ (2015). 2015 ESC Guidelines for the management of infective endocarditis: The Task Force for the Management of Infective Endocarditis of the European Society of Cardiology (ESC). Endorsed by: European Association for Cardio-Thoracic Surgery (EACTS), the European Association of Nuclear Medicine (EANM). Eur Heart J.

[10] Tuomanen EI, Austrian R, Masure HR (1995). Pathogenesis of pneumococcal infection. N Engl J Med.

[11] Tong SC, Davis JS, Eichenberger E, Holland TL, Fowler VG Jr (2015). Staphylococcus aureus infections: epidemiology, pathophysiology, clinical manifestations, and management. N Engl J Med.

[12] Ho JE, Zern EK, Lau ES (2020). Exercise pulmonary hypertension predicts clinical outcomes in patients with dyspnea on effort. J Am Coll Cardiol.

[13] Lockhart PB, Brennan MT, Sasser HC, Fox PC, Paster BJ, Bahrani-Mougeot FK (2008). Bacteremia associated with toothbrushing and dental extraction. Circulation.

[14] Lockhart PB, Brennan MT, Thornhill M, Michalowicz BS, Noll J, Bahrani-Mougeot FK, Sasser HC (2009). Poor oral hygiene as a risk factor for infective endocarditis-related bacteremia. J Am Dent Assoc.

[15] Thornhill MH, Dayer MJ, Forde JM, Corey GR, Chu VH, Couper DJ, Lockhart PB (2011). Impact of the NICE guideline recommending cessation of antibiotic prophylaxis for prevention of infective endocarditis: before and after study. BMJ.

[16] Thornhill MH, Jones S, Prendergast B, Baddour LM, Chambers JB, Lockhart PB, Dayer MJ (2018). Quantifying infective endocarditis risk in patients with predisposing cardiac conditions. Eur Heart J.

[17] Wang A, Athan E, Pappas PA (2007). Contemporary clinical profile and outcome of prosthetic valve endocarditis. JAMA.

[18] Baddour LM, Epstein AE, Erickson CC (2010). Update on cardiovascular implantable electronic device infections and their management: a scientific statement from the American Heart Association. Circulation.

[19] Hubers SA, DeSimone DC, Gersh BJ, Anavekar NS (2020). Infective endocarditis: a contemporary review. Mayo Clin Proc.

[20] Delahaye F, M'Hammedi A, Guerpillon B (2016). Systematic search for present and potential portals of entry for infective endocarditis. J Am Coll Cardiol.

[21] Cahill TJ, Baddour LM, Habib G (2017). Challenges in infective endocarditis. J Am Coll Cardiol.

